# Effect of Viewing Angle While Tightening on Torque Values in Beam-Type Torque Wrenches

**DOI:** 10.3390/dj14080464

**Published:** 2026-07-30

**Authors:** Matan Barkan, Yaniv Skvirsky, Ilan Gilboa, Dror Aizenbud, Eli E. Machtei, Zvi Gutmacher

**Affiliations:** 1 Department of Prosthodontics, School of Graduate Dentistry, Rambam Health Care Campus, Haifa 3525408, Israel; yanivskvir@gmail.com (Y.S.); gilboadr@gmail.com (I.G.); 2 Orthodontics and Craniofacial Anomalies, Oral Biology Research Laboratory, Faculty of Medicine, Technion-Israel Institute of Technology, Haifa 3525433, Israel; d_aizenbud@rambam.health.gov.il; 3 Faculty of Medicine, Technion-Israel Institute of Technology, Haifa 3525433, Israel; machtei@technion.ac.il (E.E.M.); z_gutmacher@rambam.health.gov.il (Z.G.)

**Keywords:** torque wrench, beam type, mechanical complication, prosthetic screw, screw fracture, dental implants

## Abstract

**Background/Objectives**: Controlled tightening force of implant prosthetic screws is achieved using dental torque wrenches. Beam-type wrenches are prone to a parallax error, where different viewing angles can unintentionally alter the applied force, potentially leading to screw loosening and fracture. The objective of this study was to determine the effect of different viewing angles of the operator relative to the wrench’s reading scale and the actual force applied on the prosthetic screw. **Methods**: Two types of beam torque wrenches (Institut Straumann AG, Switzerland; MIS Implant Technologies, Israel) were used. Five wrenches for each group were connected to a torque meter mounted on a custom 3D printed apparatus, allowing torque read at different viewing angles. Each wrench was tightened according to manufacturer recommendations at angles ranging between ±60 degrees from center position. A camera mounted on a tripod provided magnification and consistency in recordings. For each measurement, a comparison was made between intended tightening force and actual force exerted on the torque meter. Data were analyzed to determine the effect on clinical settings. **Results:** Both wrenches exhibited a parallax effect with its resulting impact on force delivery by the wrench. When the operator was positioned at a negative angle relative to the wrench, while applying tightening force, the actual force delivered by the wrench was amplified. The greater the angle, the greater the force amplification was. The opposite effect took place when the wrench was viewed from a positive angle relative to the operator. Force alteration reached up to 8.72 Ncm, which can account for as much as 25% from intended force. This effect was notable and statistically significant for both types of wrenches. **Conclusions**: The tested beam-type torque wrenches present an inherent potential deficiency in which oblique viewing angles lead to either amplification or attenuation of the actual force that is applied. This phenomenon might be associated with a variety of prosthetic failures. Operators are recommended to adopt a viewing strategy that minimizes undesired outcomes.

## 1. Introduction

Dental implants have become a widely accepted and used approach in prosthetic dentistry. Although tooth loss is in a state of decline, the need for treatment is in constant rise [1].

A prosthetic screw is the most widespread method of connecting and securing abutments, crowns and prosthetic attachments to the implant itself [2,3,4]. Each implant system and manufacturer has a recommended optimal torque value for screw closure. Deviation from desired torque values can have undesirable implications: over tightening can result in screw fracture, which is considered a fatal failure. Under tightening of the prosthetic screw can result in screw loosening, which in turn can lead to screw deformation or fracture [2,4,5,6,7,8,9]. Tightening to a specific torque value is achieved using a specialized torque wrench that engages the prosthetic screwdriver and is designed to control the applied force. The two common types of wrenches are friction-style and beam-style wrenches. In a friction-style wrench, the clinician pre-sets the desired torque value; once the desired torque level is reached, the handle’s joint collapses, thus halting further force application. The beam-style wrench lacks an automatic release mechanism. Instead, torque values are marked on the device handle. The operator applies force to the beam that is attached to the handle until the beam aligns with the marking that denotes the required torque [10]. No current consensus exists that suggests which wrench type is favorable. Some studies were not able to find significant differences in performance between the two types [11,12]. Other studies have suggested that beam-type devices may provide greater accuracy than friction-type devices [12,13] and offer greater consistency, which may contribute to improved accuracy in clinical practice [14]. To the contrary, others have reported better results for friction-style wrenches [15].

Parallax is an optical phenomenon in which the apparent position or direction of an object changes when viewed from different angles [16]. This effect may occur when a beam-type wrench is not observed exactly perpendicular to both the beam and the marker arm. Additionally, the parallax effect was found to cause reading errors on other measurement gauges, not necessarily related to dentistry [17], and to have a negative impact on general reading speed [18]. The magnitude of the parallax error has been shown to depend not only on the viewing position, but also on the dimensions and configuration of the beam-type wrench [19].

This phenomenon was investigated in previous studies [20] which reported an association between viewing angle and variations in force delivery. Wadhwani et al. [19] has made precise measurements using a digital camera to study the effect of the operator’s viewing angle on the measurement error and verified the existence of an angle related reading error. However, in that article, only one wrench was tested and without the use of a torque meter. Yaylaci et al. [21] studied the parallax effect on a beam-type wrench using a torque meter and demonstrated discrepancies in force activation at different angles, but did not use a digital camera and only tested from a few viewing angles (0, 30 and 90 degrees).

Thus, the purpose of the present study is to examine and compare two different beam-type torque wrenches under different viewing angles when attempting to apply a tightening force. The null hypothesis is that the magnitude of the parallax effect will not differ between the different devices.

## 2. Materials and Methods

This in vitro study compared the torque delivered by beam-type torque wrenches from two manufacturers across a range of operator viewing angles. The concept and methodological basis of the study were derived from previously published work examining the parallax effect in beam-type torque wrenches [19,21], whose protocols informed the design of the present experiment.

**Figure 1:**
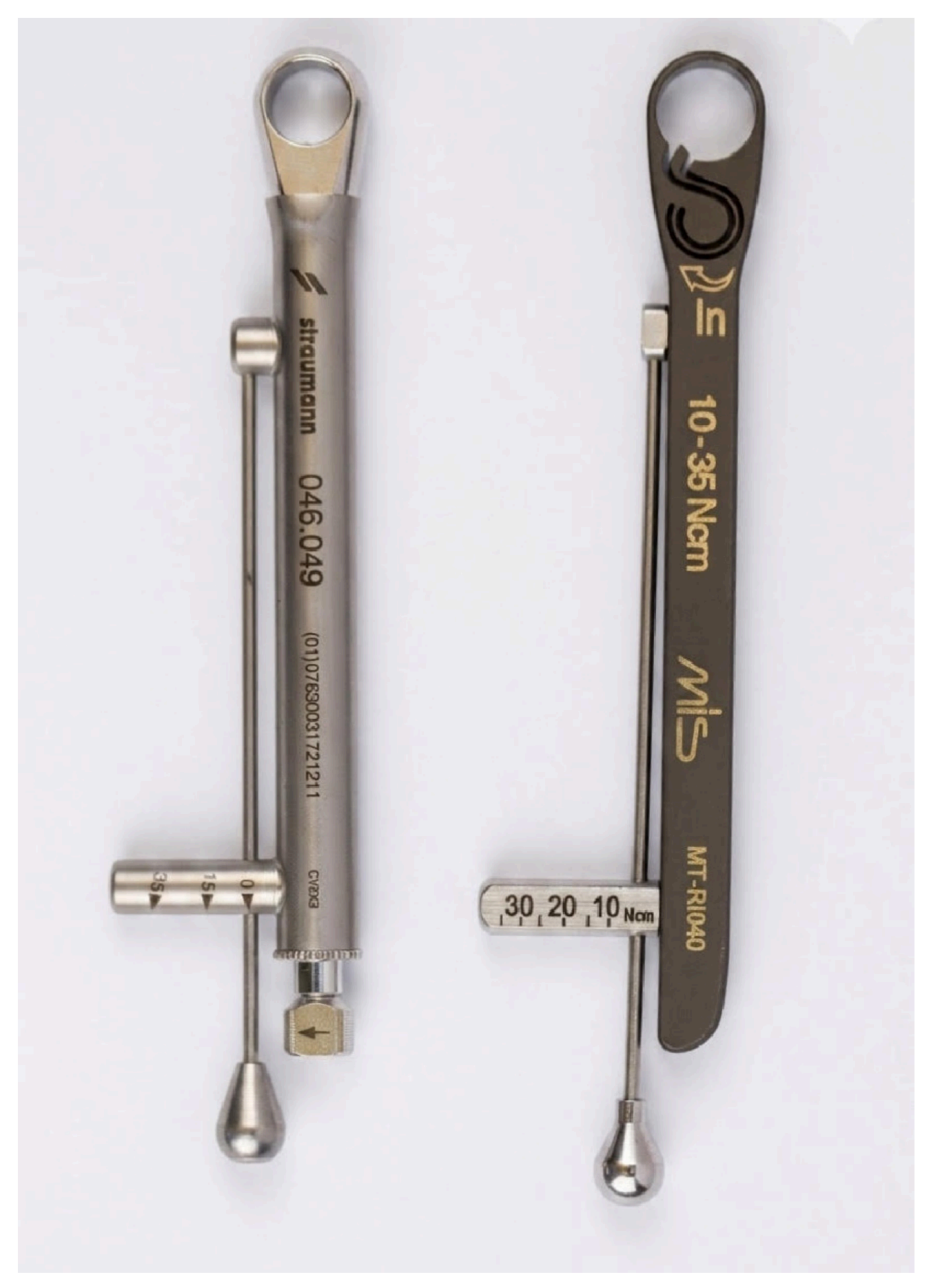
The two types of beam-type wrenches being tested. MIS (**right**), STR (**left**).

New, unused wrenches were used. These included the Straumann^®^ Torque Control Device for Ratchet, ref. 046.049 (Institut Straumann AG, Basel, Switzerland; hereafter STR) and the MIS^®^ MT-RI040 Prosthetic Torque Ratchet (MIS Implants Technologies Ltd., Bar-Lev Industrial Park, Israel; hereafter MIS), with five separate wrenches tested from each manufacturer (Figure 1). Each wrench constituted an independent sampling unit: every viewing angle was measured once on each of the five wrenches, such that n = 5 per angle per manufacturer represented five different devices rather than repeated trials on a single device. Each wrench was connected to its original matching screwdriver, which was fixed to a torque meter with a stated accuracy of 1.5% and a resolution of 0.1 Ncm (TQ-8800; Lutron Electronic Enterprise Co., Ltd., Taipei City, Taiwan).

**Figure 2:**
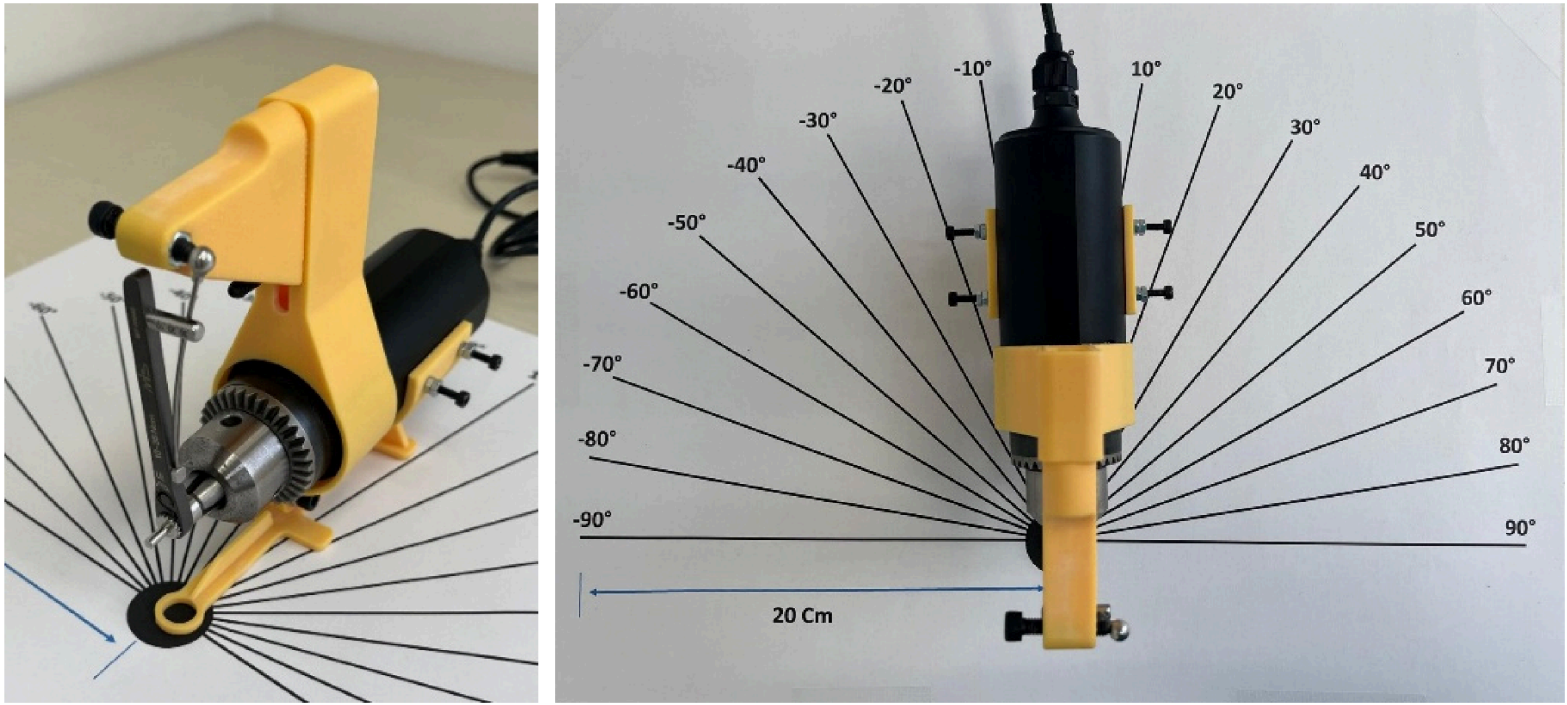
A custom-designed, 3D-printed apparatus enabling control of viewing angles, force application and torque reading.

**Figure 3:**
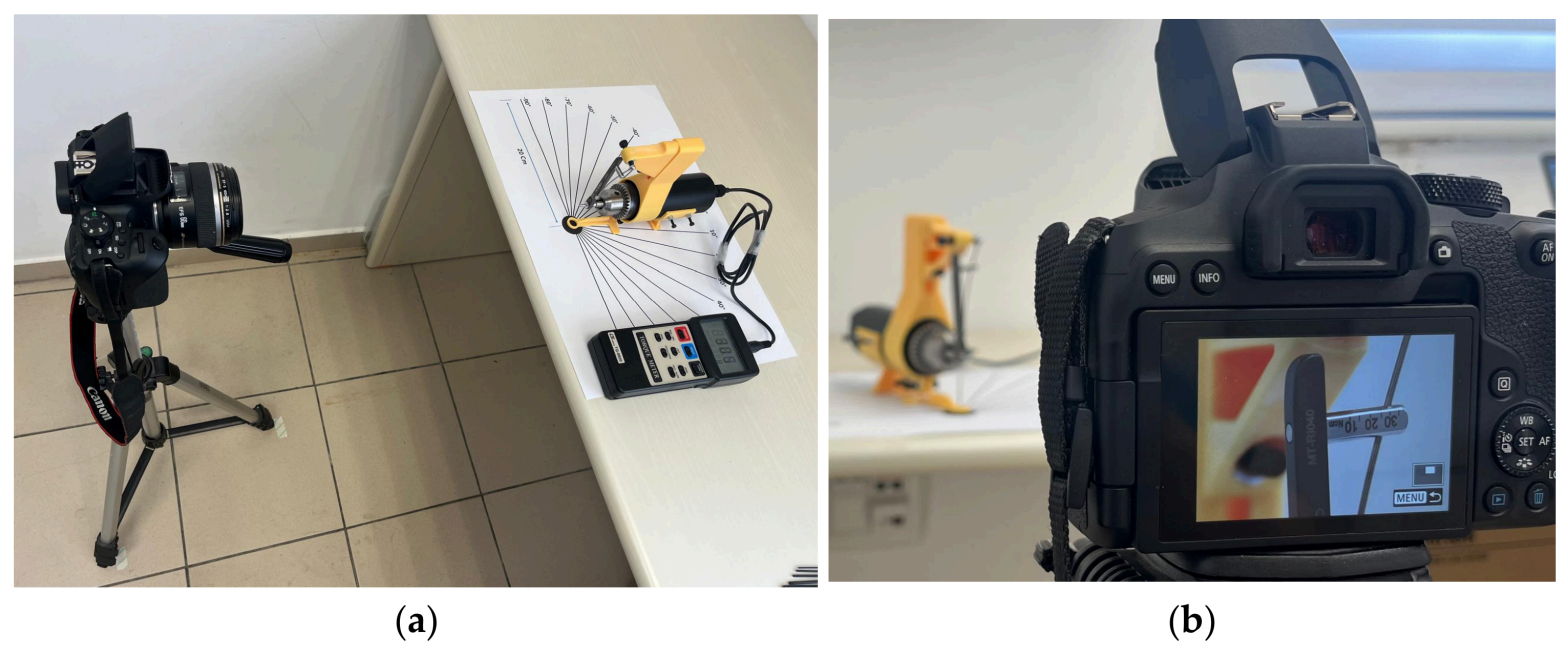
The set up: (**a**)—the camera mounted on a tripod at a working distance of 35 cm from the wrench. (**b**)—demonstration of the DSLR camera point of view, assisting in precise force activation at every viewing angle.

The torque meter was mounted on a custom 3D-printed apparatus, designed by author Y.S., which permitted controlled variation in the operator’s horizontal viewing angle relative to the wrench and thereby simulated the intraoperative viewing angle (Figure 2). To reproduce the operator’s line of sight and working distance, a digital SLR camera (850D with a 60 mm lens; Canon Inc., Tokyo, Japan) was mounted 35 cm from the screwdriver position [22], in line with the 0° marker; the camera image was used to verify the value observed on the wrench scale at the moment tightening force was applied (Figure 3).

Five measurements were obtained for each condition, each performed with a different wrench, yielding 65 measurements for STR and 45 for MIS (110 in total). The MIS wrench was tightened to 30 Ncm and the STR wrench to 35 Ncm, in accordance with the respective manufacturers’ recommendations. Tightening was performed at successive viewing angles in 10° increments on the horizontal plane: the STR wrench was tested from −60° to +60°, whereas the MIS wrench was tested only from −40° to +40°, owing to a physical limiter inherent to its design. The torque value delivered at each angle was measured and recorded by the torque meter, and all measurements were performed on the same day by a single operator (M.B.).

For the statistical analysis, a linear mixed-effects model was used to evaluate the relationship between the delivered torque and the viewing angle. The dependent variable was the centered measurement, defined as the deviation of the delivered torque from the manufacturer’s target value (30 Ncm for MIS, 35 Ncm for STR), such that zero represents exact agreement with the target. Standardized angle, manufacturer (MIS or STR), and their interaction were entered as fixed effects, and a random intercept and a random slope for standardized angle were specified for each individual device to account for repeated measurements taken on the same wrench. Angle values were standardized across the dataset. Statistical significance was set at *p* < 0.05, and a 95% confidence level was used for all analyses.

## 3. Results

**Figure 4:**
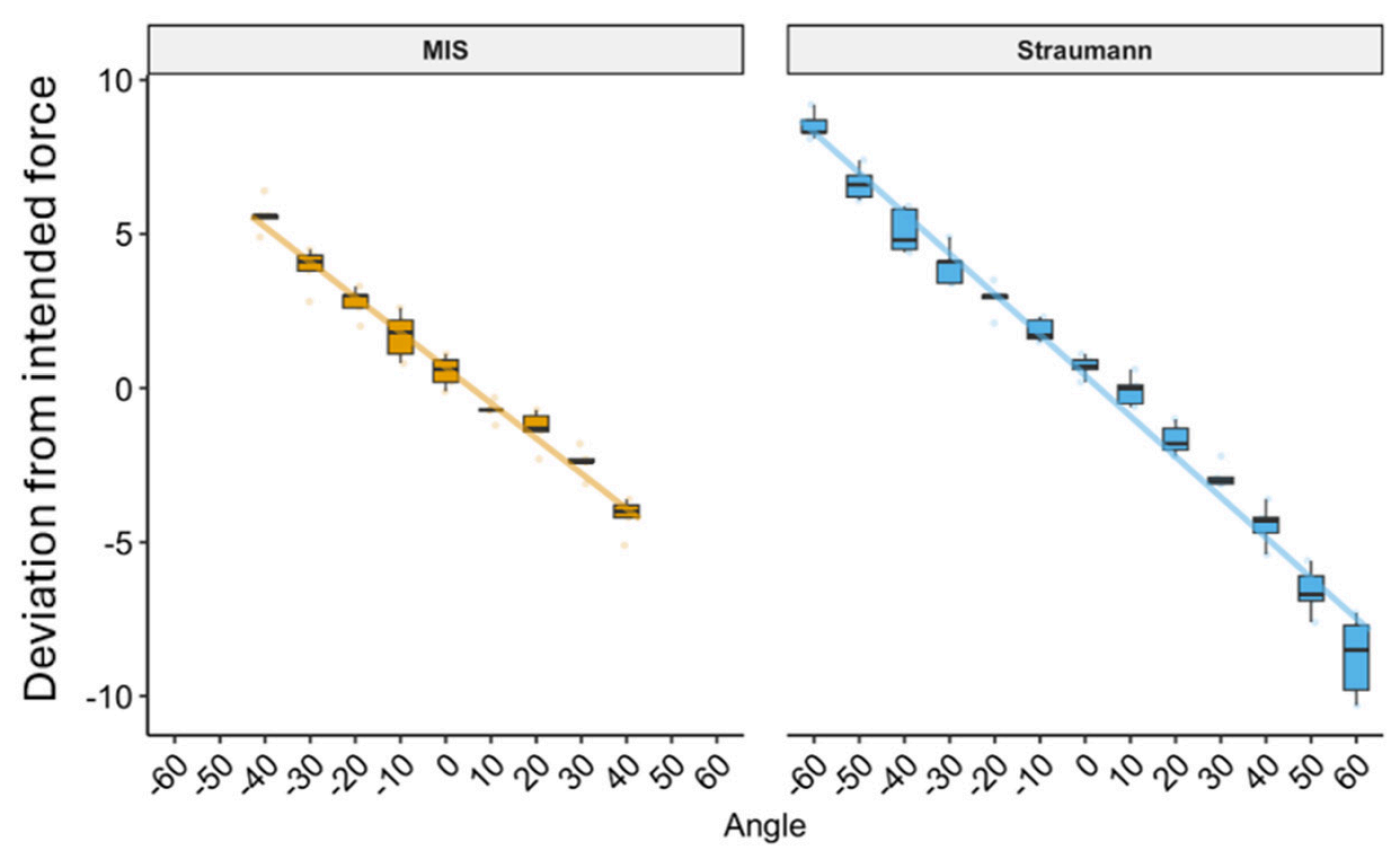
Box plot showing deviation from the intended force relative to viewing angle for MIS (**left**) and Straumann (**right**).

A total of 110 measurements were obtained, with five repeated measurements recorded at each perpendicular and angular reading for both devices (Table 1). As the deviation in viewing angle from the zero position was increased, greater mean deviation from the intended force was recorded (Figure 4). In the MIS group, mean deviation values ranged from +5.60 Ncm (±0.53) at −40° to −4.14 Ncm (±0.58) at +40°. In the Straumann group, mean deviation values ranged from +8.52 Ncm (±0.44) at −60° to −8.72 Ncm (±1.30) at +60° viewing angle. At comparable angles of −40° and +40°, the Straumann group showed mean deviation values of +5.08 Ncm (±0.72) and −4.44 Ncm (±0.67), respectively. It is also worth noting that even at a 0 degree there is a slight deviation from the targeted torque (0.7 ± 0.34 and 0.54 ± 0.49 Ncm) for the STR and MIS wrenches respectively. Within all viewing angles, there were relatively small deviations between the repeated measurements, with SD values generally below 1.0 for both devices.

Linear mixed-effects analysis demonstrated a significant predictor relationship between viewing angle and force deviation: the greater the angle was (relative to the zero position), the greater the deviation in the actual force that was delivered (*p* < *0*.001). The device manufacturer effect was found not statistically significant (*p* = 0.316). However, the interaction between standardized angle and device manufacturer was significant (*p* = 0.021), indicating that the relationship between angulation and measurement differed between the manufacturers. Overall, both devices showed a consistent linear reduction in centered measurement as angulation increased, with STR exhibiting a slightly steeper incline compared with MIS (Figure 4).

**Figure 5:**
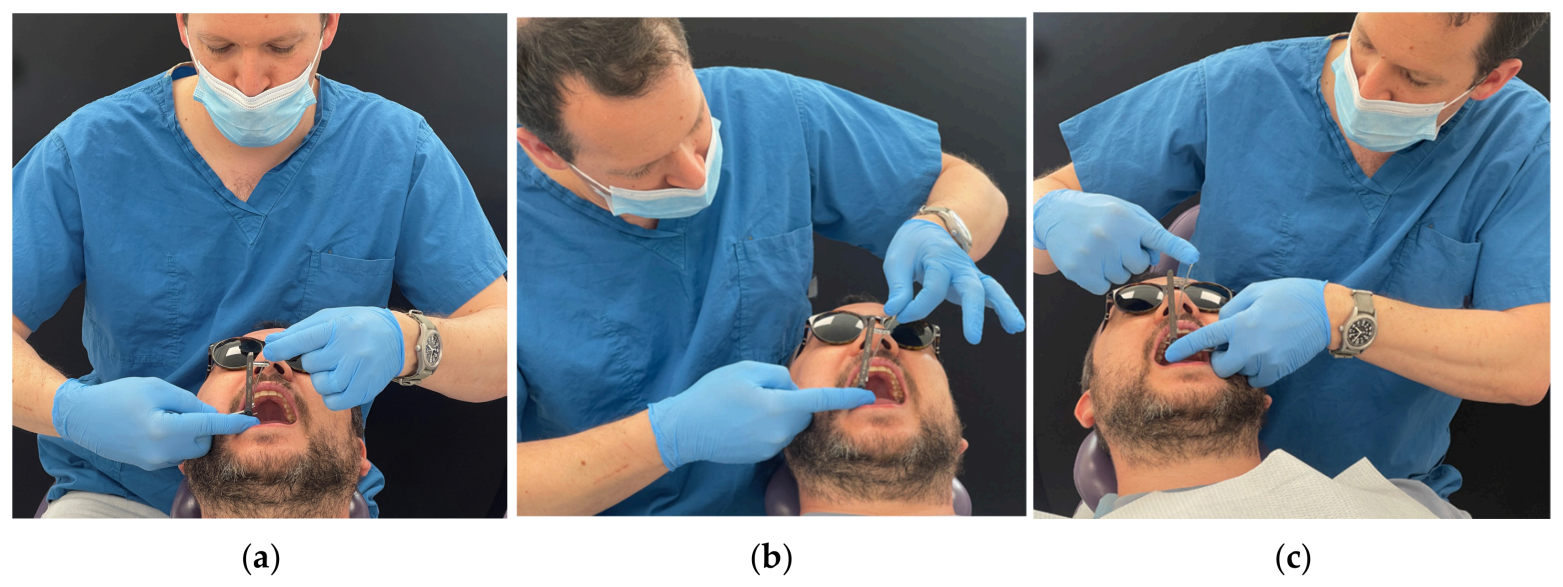
A demonstration of the position of the operator relative to the torque wrench in clinical settings: (**a**)—viewing from the positive side. (**b**)—viewing from the zero/straight position. (**c**)—viewing from the negative side.

Greater deviation from the zero position results in a proportional deviation of the applied force. When viewing the wrench from the negative angle side (left-hand side when working on the lower jaw, right-hand side when working on the upper jaw) (Figure 5), the actual force is being amplified, while when viewing from the positive angle side (right-hand side when working on the lower jaw, left-hand side when working on the upper jaw), the actual force is being reduced (+5.08 to −4.44 and +5.6 to −4.14 for the STR and MIS respectively at ±40°). For each incremental deviation in viewing angle, a corresponding deviation in actual force was observed.

## 4. Discussion

Beam-type torque wrenches rely on the operator’s visual alignment of the beam with a reading scale, making them susceptible to parallax error. The aim of this study was to quantify the effect of the operator’s viewing angle on the actual torque delivered by two beam-type wrenches. Viewing angle was found to have a significant effect on the applied force, with deviations of up to 8.72 Ncm—approximately 25% of the intended torque. This phenomenon was observed in all wrenches from both groups, and in a similar manner; thus, the null hypothesis was rejected. Shiba et al. [20] has demonstrated the parallax effect’s influence on beam-type wrenches, where accuracy between intended and actual force application was high at the zero position, but diminished significantly at viewing angles of 60 and 30 degrees (other angles were not reported). Likewise, Yaylaci et al. [21] also reported changes in torque values at different viewing angles—after testing beam-type wrenches at 30, 60 and 90 degree viewing angles, it was shown that maximum force deviation occurs at the greatest angle. While the angle measurement system was different in said studies (i.e., determining what is the “zero” position in terms if degrees), the findings show a similar trend, whereby deviation from the ‘zero’ viewing angle leads to distortion in the actual force applied.

In the current study, both wrenches (being of the same design category) showed comparable results. Several studies were designed to compare different types of dental torque wrenches and their accuracy; Vallee et al. [13] and Rajatihanghi et al. [23] showed that when compared together, beam-type wrenches exhibited less deviation than friction-type wrenches. In contrast, Albayrak et al. [24] showed no statistically significant difference between the two types of wrenches while Skvirsky et al. [10] showed better trueness and precision for friction-type wrenches. The similarities in results for the two wrenches in our study could be attributed to the similarities in shape and size, design, material and operating mechanism. It is worth mentioning again that the MIS wrench features a physical limiter restricting the beam from extreme positions; thus, a comparative analysis for the more extreme angles (50–60°) was not feasible.

In the present study, off-centric viewing angles could have led to an over-tightening of up to 8.52 Ncm and under-tightening of −8.72 Ncm which are dependent on the operator’s position; such a phenomenon could lead to prosthetic failure. Kose [25] showed that insufficient tightening or pre-load can lead to early screw loosening and even fracture. Sang- Choon Cho [26], in a 3 to 7 year longitudinal study of dental implants, reported a prevalence of 14.5% and 5.8% screw loosening during this period (for standard and wide diameter implants, respectively). Likewise, Katsavochristou [27], in a systematic review, reported that abutment screw loosening incidents ranged between 7 and 11%, while abutment screw fracture incidents were found to be at 0.6%. To the contrary, Theoharidou [28], in a systematic review, showed that abutment screw loosening is a rare event in a single implant restoration regardless of the geometry of the implant–abutment connection, provided that proper torque and anti-rotational features are employed. Over tightening is mentioned in the literature as a possible cause of screw fracture [29]; however, controlled studies designed to examine this effect are scarce and this phenomenon will require future research.

The clinical relevance of these deviations should be considered. A recent meta-analysis concluded that output torque values within 10% of the target are clinically acceptable for prosthetic screw tightening [14]. Applying this threshold (±3.0 Ncm for MIS, ±3.5 Ncm for STR), deviations recorded at viewing angles of up to ±20° remained within clinically acceptable limits, whereas angles of 30° and beyond produced deviations exceeding this threshold—reaching approximately 25% of the intended torque at the most extreme positions. Thus, the parallax effect becomes clinically meaningful primarily at moderate-to-large angular deviations, which may, nonetheless, be encountered in routine intraoral working positions.

A possible limitation of this study lies in the fact that each angle was measured only once for each wrench and by a single operator; this might not have accounted for the inherent discrepancies in accuracy and precision that exist between wrenches and between operators, as shown by Skvirsky et al. [10] and Goyal et al. [30], which may have further influenced the results. A related limitation concerns the sample size: testing was performed on five wrenches per manufacturer, a number that could not be increased, as it was constrained by the wrenches available for evaluation. A larger sample would improve the statistical power of the analysis and better capture between-wrench variability within each manufacturer, and the present findings should be interpreted with this in mind. Moreover, only two groups of beam-type wrenches were tested, and evaluating additional devices may yield similar or different results. Future studies should therefore employ a larger number of wrenches per group, ideally across multiple production batches, and evaluate a broader range of beam-type wrenches with different beam lengths, scale designs, and marker configurations, as these geometric factors may modulate the magnitude of the parallax error, together with clinically relevant variables such as operator experience and the use of magnification loupes.

This study investigated a seemingly inherent limitation of beam-type wrenches affecting their use. Conversely, friction-type wrenches do not feature this limitation because using a friction-type wrench is not dependent on the operator’s feedback or viewing angle. However, as stated previously, these wrenches may be less accurate under certain circumstances due to other modifying factors [10].

## 5. Conclusions

Within the limitations of this in vitro study, the following results were ascertained:

1. The operator’s viewing angle significantly affected the force delivered by beam-type torque wrenches: negative angles amplified the applied torque, while positive angles attenuated it, with deviations of up to 8.72 Ncm (approximately 25% of the intended force).
2. Both devices exhibited this parallax effect in a comparable manner, suggesting an inherent limitation of the beam-type design rather than a manufacturer-specific deficiency.
3. While small angular deviations remained within clinically acceptable limits, larger angles produced discrepancies that may contribute to screw loosening or fracture.
4. Clinicians are therefore advised to position their line of sight perpendicular to the reading scale to minimize this error and improve torque accuracy.

## Figures and Tables

**Table 1:** Mean values and standard deviations from the intended force for MIS and Straumann wrenches.

Angle	MIS Mean	±SD	STR Mean	±SD
−60	-	-	8.52	0.44
−50	-	-	6.64	0.53
−40	5.6	0.53	5.08	0.72
−30	3.9	0.67	3.98	0.62
−20	2.78	0.5	2.9	0.5
−10	1.7	0.75	1.86	0.36
0	0.54	0.49	0.7	0.34
10	−0.72	0.32	−0.08	0.49
20	−1.32	0.62	−1.66	0.5
30	−2.4	0.46	−2.86	0.38
40	−4.14	0.58	−4.44	0.67
50	-	-	−6.58	0.77
60	-	-	−8.72	1.3

## Data Availability

The original data presented in the study are openly available in https://figshare.com/articles/dataset/Data_for_article_-_Effect_of_viewing_angle_while_tightening_on_torque_values_in_beam_type_torque_wrenches/32515422?file=65108964, 12 April 2026.
